# Rational design and microwave-promoted synthesis of triclosan-based dimers: targeting InhA for anti-mycobacterial profiling

**DOI:** 10.1098/rsos.240676

**Published:** 2024-10-09

**Authors:** Shekhar, Francoise Roquet-Banères, Amit Anand, Laurent Kremer, Vipan Kumar

**Affiliations:** ^1^ Department of Chemistry, Guru Nanak Dev University, Amritsar, Punjab 143005, India; ^2^ Centre National de la Recherche Scientifique UMR 9004, Institut de Recherche en Infectiologie de Montpellier (IRIM), Université de Montpellier, 1919 route de Mende, 34293 Montpellier, France; ^3^ Department of Chemistry, Khalsa College, Amritsar, Punjab 143005, India; ^4^ INSERM, IRIM, 34293 Montpellier, France

**Keywords:** triclosan, click chemistry, anti-mycobacterial, InhA, *M. abscessus*

## Abstract

A set of alkyl-/1H-1,2,3-triazole-based dimers was strategically designed and synthesized to evaluate their *in vitro* anti-mycobacterial activities against *Mycobacterium tuberculosis* and the non-tuberculous *Mycobacterium abscessus* strains. Systematic variations in the nature (alkyl/1H-1,2,3-triazole) and positioning of the linker were implemented based on the docking scores observed in the binding sites identified in the crystal structures of InhA from *M. tuberculosis* and *M. abscessus*. However, the *in vitro* evaluation results revealed that the synthesized compounds did not exhibit inhibitory effects on the growth of mycobacteria, even at the highest tested concentrations. The elevated lipophilicity values determined through ADMET studies for these synthesized dimers might be a contributing factor to their poor activity profiles.

## 1. Introduction

Tuberculosis (TB) is an infectious disease caused by the highly virulent microorganism *Mycobacterium tuberculosis* (*Mtb*). Transmission of disease occurs when individuals suffering from TB release bacteria into the air through coughing and sneezing. Each year, over 10 million individuals fall ill with TB worldwide. In 2022, an estimated 10.6 million people developed TB, reflecting an increase from the best estimates of 10.3 million in 2021 and 10.0 million in 2020. TB emerged as the second-leading cause of death worldwide attributable to a single infectious agent, surpassed only by the impact of coronavirus disease (COVID-19) in 2022. Notably, TB accounted for nearly twice as many fatalities as HIV/AIDS [1]. The treatment of TB is a tedious, challenging and prolonged process. The current standard regimen for treating drug-sensitive TB involves a combination of four primary drugs (isoniazid, rifampicin, pyrazinamide and ethambutol) administered over six months through directly observed therapy with subsequent support. However, prolonged use of multiple medications carries the risk of adverse reactions, potentially leading to the discontinuation of anti-TB treatment and the development of more severe forms like multidrug-resistant TB and extensively drug-resistant TB. There is a growing concern that TB may once again become an incurable illness due to the prevalence and severity of drug-resistant strains [2–4]. Therefore, there is an urgent need for the development and more efficient evaluation of new TB drugs and shorter treatment regimens.

**Figure 1.**
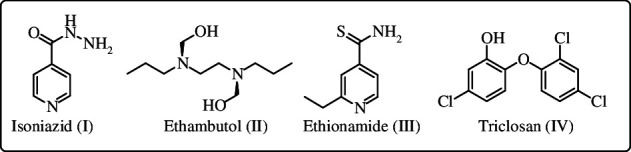
Structures of first-line drug isoniazid (I), ethambutol (II), second-line drug ethionamide (III) and triclosan (IV).

The enoyl-acyl carrier protein reductase, commonly known as *M. tuberculosis* InhA, presents itself as a promising target for the development of novel anti-tuberculosis drugs [5]. Isoniazid (INH) (figure 1), a primary drug in current TB treatment regimens, was introduced in 1952. However, its antitubercular action was only recently correlated to the inhibition of InhA that participates in mycolic acid biosynthesis. INH acts as a pro-drug, necessitating enzymatic activation through the catalase-peroxidase KatG. This activation leads to the formation of an isonicotinoyl radical, which then forms a covalent INH–NAD adduct. This adduct, in turn, serves as a potent inhibitor of InhA [6–9]. Ethionamide (ETH) (figure 1), a second-line antitubercular drug, also targets InhA. This agent, however, is activated by a different enzyme, flavin monooxygenase (EthA) [10,11]. Mutations in the corresponding enzymes, KatG and EthA, that activate INH and ETH, respectively, are the main cause of resistance to these drugs in *Mtb* [12,13]. Hence, the development of direct inhibitors targeting InhA becomes a promising strategy for tackling *Mtb* strains resistant to ETH and INH, circumventing the requirement for KatG or EthA activation [14,15].

Triclosan (TCS) (figure 1) is a widely used antimicrobial agent present in disinfectants, soaps, detergents, toothpastes, mouthwashes, fabrics, deodorants, shampoos and plastic additives, in addition to innumerable other personal care, veterinary, industrial and household products [16,17]. It is recognized as a promising compound for inhibiting InhA, a validated and attractive anti-TB target. This polychlorinated molecule does not require any bioactivation by KatG and it is able to directly affect the function of InhA [18]. It was proven to inhibit InhA at sub-micromolar concentration range [15]. Tailoring of the TCS scaffold resulted in the synthesis of a wide range of direct inhibitors of this enzyme [19–21]. While TCS serves as a moderate inhibitor for InhA, it serves as the cornerstone for numerous studies that utilize its scaffold in framing more potent inhibitors [15,17,20].

Recently, we have disclosed the synthesis and anti-mycobacterial evaluation of TCS based azo-adducts against both TB and non-TB mycobacteria. Their mode of action was unravelled by analysing the InhA overexpression, cross-resistance determination and conducting biochemical analyses. These compounds effectively inhibit mycolic acid biosynthesis and do not necessitate the KatG-mediated activation step [22–26]. The present focus of this work therefore specifically aimed at developing direct inhibitors of InhA, which does not require pre-activation by KatG. In continuation, this study includes the design, syntheses and characterization of a new series of TCS dimers along with their anti-mycobacterial evaluation.

## 2. Results and discussion

In our efforts to find direct inhibitors for mycobacterial InhA, we designed a series of TCS dimers. These dimers feature two TCS molecules linked together by alkyl linkers of varying chain length. The design of these compounds was geared towards occupying the binding sites identified in the crystal structures of InhA from *M. tuberculosis* (PDB: 1P45) and InhA from *M. abscessus* (PDB: 7U0M).

**Figure 2.**
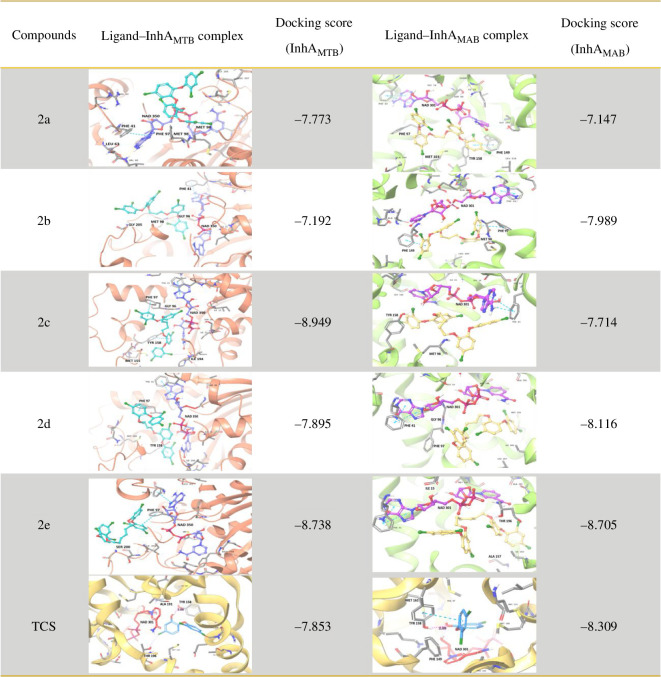
Predicted binding modes of TCS dimers at the active site of InhA_MTB_ (PBD ID: 1P45) and InhA_MAB_ (PBD ID: 7U0M). Residues are shown with grey carbons, NAD^+^ is shown with purple and pink carbons, respectively, dimers are shown with turquoise and yellow carbons, respectively. TCS is represented with sky-blue carbons.

Scrutinizing the protein–ligand interactions of these dimers (figure 2) with InhA_MTB_, we observed intriguing halogen bond and π–π stacking interactions. Specifically, halogen bonds formed between various chlorines of TCS and amino acids such as MET-98, ILE-194 and TYR-158. Moreover, notable π–π stacking interactions emerged between the aromatic rings of TCS and amino acids PHE-97 and TYR-158. Remarkably, the docking scores for these ligands ranged from −7.192 to −8.949, surpassing that of TCS, which scored −7.853. Conversely, halogen bond interactions were noted between TCS chlorines and amino acids MET-103, TYR-158, MET-98, GLY-96 and THR-196 within the binding pocket of InhA_MAB_. Additionally, π*–*π stacking interactions were observed between the TCS ring and amino acids PHE-149 and PHE-97. The docking scores for these interactions fell within a comparable range of −7.147 to −8.705, similar to that of TCS (−8.309).

Based on our docking analysis, we deduced that TCS dimers could effectively fit into the active site of InhA for both mycobacterial species, indicating potential selectivity for InhA. This insight guided our designed approach, where we joined two TCS molecules using a symmetrical and flexible alkyl linkage. Our strategy revolved around the notion that a longer and more flexible chain linker could facilitate the enlarged molecule to adopt a conformation similar to that required in the active site [20]. We synthesized, characterized and assessed five TCS dimers for their anti-mycobacterial activities.

**Scheme 1.**
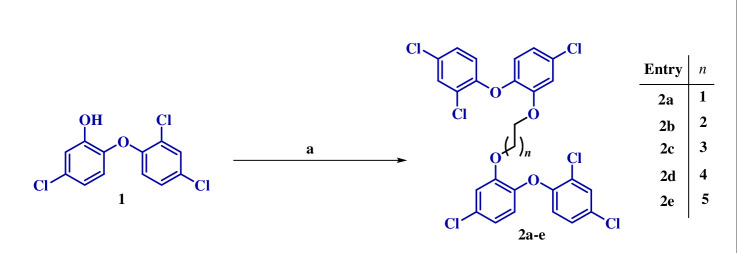
(a) K_2_CO_3_, dibromoalkane, DMF, 100°C, 20 min, microwave.

The synthetic methodology involved the base-promoted generation of phenolate anion of TCS (**1**) followed by the addition of dibromoalkanes to yield the corresponding dimer (**2a**–**e**) as shown in Scheme 1. The structures of the synthesized dimers were confirmed based on the spectral data and analytical evidence. For instance, compound **2b** exhibited a molecular ion peak at *m*/*z* C_27_H_18_Cl_6_O_4_ [M]^+^ 615.9336, [M + 2]^+^ 617.9307, [M + 4]^+^ 619.9277 and [M + 6]^+^ 621.9248 in its high-resolution mass spectrum. Its ^1^H NMR spectrum displayed the presence of doublets at *δ* 7.40, 6.85 and 6.53 indicating aromatic protons of TCS. The presence of a triplet and multiplet at *δ* 3.80 and 1.98–1.89, respectively, indicated the –CH_2_ of alkyl linkers. Additionally, ^13^C NMR spectrum further corroborated the assigned structure having all the necessary number of carbons, including characteristic peaks at *δ* 152.6 and 114.75 corresponding to aromatic carbons of TCS whereas peaks at *δ* 64.59 and 28.79 indicated the aliphatic carbons of –CH_2_ linker.

The synthesized TCS dimers (**2a**–**e**) were then evaluated for their anti-mycobacterial activities against the smooth variant of *M. abscessus* (*Mabs*) CIP104536^T^ and *M. tuberculosis* (*Mtb*) strain mc^2^6230 using TCS, rifabutin (RFB) and INH as references, and the corresponding minimum inhibitory concentration (MIC) values are listed in table 1. As evident from the data, the synthesized TCS dimers failed to inhibit the growth of mycobacteria at the highest tested concentration, 100 µg ml^−1^.

**Table 1.** MIC values determined either in 7H9^OADC/Tylox^ broth against *M. tuberculosis* (*Mtb*) mc^2^6230 at 37°C or in cation-adjusted Mueller–Hinton broth (CaMHB) against *M. abscessus* (*Mabs*) CIP104536^T^ (S variant) at 30°C. Experiments with *M. abscessus* were done three times in duplicate and experiments with *M. tuberculosis* were done twice.

compound	MIC (µg ml^−1^)	
	*Mabs* S (CaMHB)	*Mtb* (7H9^OADC/Tylox^)
**2a**	>100	>100
**2b**	>100	>100
**2c**	>100	>100
**2d**	>100	>100
**2e**	>100	>100
TCS	6.25	2.5
INH	>10	0.03
RFB	50	0.19

After getting the unfavourable biological results, we designed another set of substituted TCS dimers containing 1H-1,2,3-triazole using click reaction. 1,2,3-Triazoles have gathered considerable interest due to their fascinating physical and biological attributes, along with their remarkable stability. The 1,3-dipolar cycloaddition reaction involving a 1,3-dipole and a dipolarophile (an alkyne) to synthesize five-membered heterocycles represents a widely recognized transformation in the field of synthetic organic chemistry [27]. Further, in medicinal chemistry, compounds containing the triazole core are attracting the attention of medicinal chemists due to their immense therapeutic potential such as antifungal [28], antiviral [29], anticancer [30], antibacterial [31], antitubercular [32] and antimalarial [33] activities.

**Figure 3.**
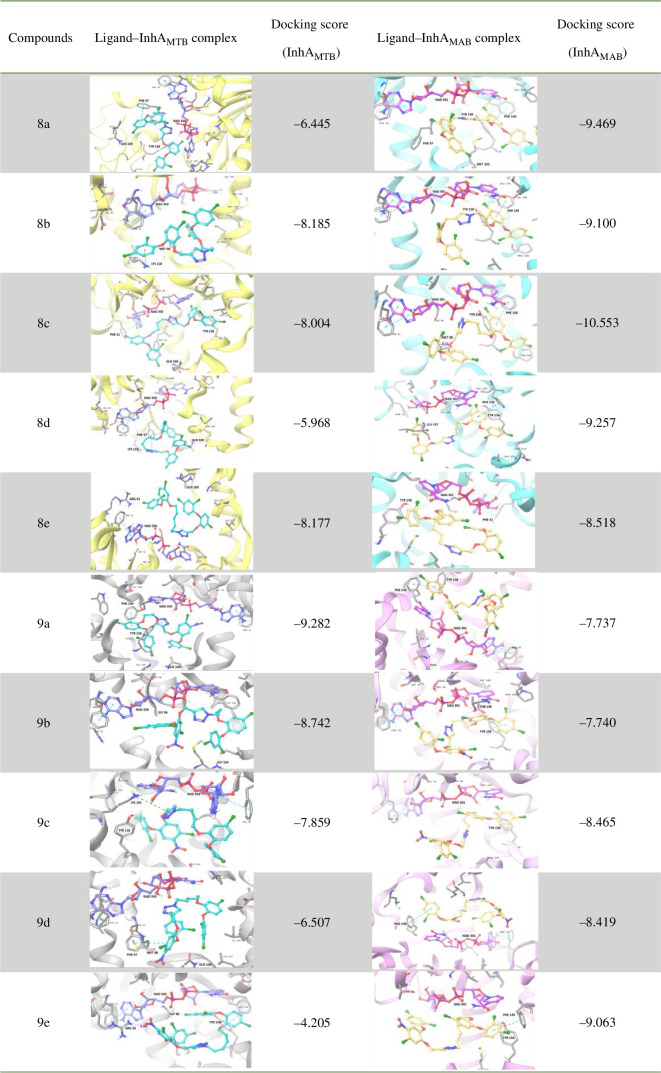
Predicted binding modes of TCS dimers at the active site of InhA_MTB_ (PBD ID: 1P45) and InhA_MAB_ (PBD ID: 7U0M). Residues are shown with grey carbons, NAD^+^ is shown with purple and pink carbons, respectively, dimers are shown with turquoise and yellow carbons, respectively.

The triazole tethered TCS dimers were initially assessed for *in silico* validation against both strains (InhA_MTB_ and InhA_MAB_). Docking results revealed superior docking scores for both strains compared with previously synthesized dimers. The docking scores for each strain are detailed in figure 3. Key interactions within the binding pocket of InhA_MTB_ included π*–*π stacking between the TCS ring and PHE-97, TYR-158 and PHE-149, as well as between the triazole ring and PHE-97 and TYR-58. Halogen bonding occurred between the chlorine of TCS and amino acids GLN-100, GLY-104, GLY-96, MET-98 and ARG-43. Cation–π interactions were also observed between the TCS ring and LYS-118, as well as between the triazole ring and LYS-165. Additionally, hydrogen bonding was observed between the oxygen of the –NO_2_ group of the molecule and the ARG-43 amino acid. In the case of InhA_MAB_, π*–*π stacking interactions were noted with amino acids PHE-149 and TYR-158, while halogen bonding occurred with TYR-158, MET-103, LEU-197, THR-158 and THR-196. Hydrogen bonding was observed between the ether oxygen of TCS and MET-98. On the basis of these docking scores we synthesized, characterized and evaluated these triazole tethered TCS dimers for their anti-mycobacterial activities.

**Scheme 2.**
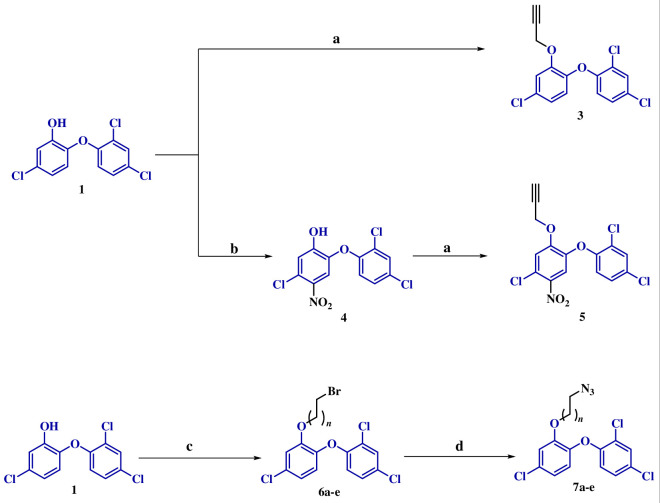
(a) Propargyl bromide, K_2_CO_3_, 60°C, 4–5 h. (b) CH_2_Cl_2_, HNO_3_, room temperature. (c) K_2_CO_3_, dibromoalkane, DMF, 100°C, 20 min, microwave. (d) NaN_3_, DMF, 120°C, 20 min, microwave.

The synthetic methodology to access these compounds involved an initial nitration of **1** in CH_2_Cl_2_ to afford precursor **4**. In separate reactions, unsubstituted TCS **1** and nitro derivative **4** were subjected to *O*-propargylation using potassium carbonate, yielding precursors **3** and **5**. Another set of precursors, *O*-alkylazido TCS (**7a**–**e**), were prepared via an initial reaction of **1** with dibromoalkane to afford **6a**–**e** with subsequent azidation with sodium azide (Scheme 2).

**Scheme 3.**
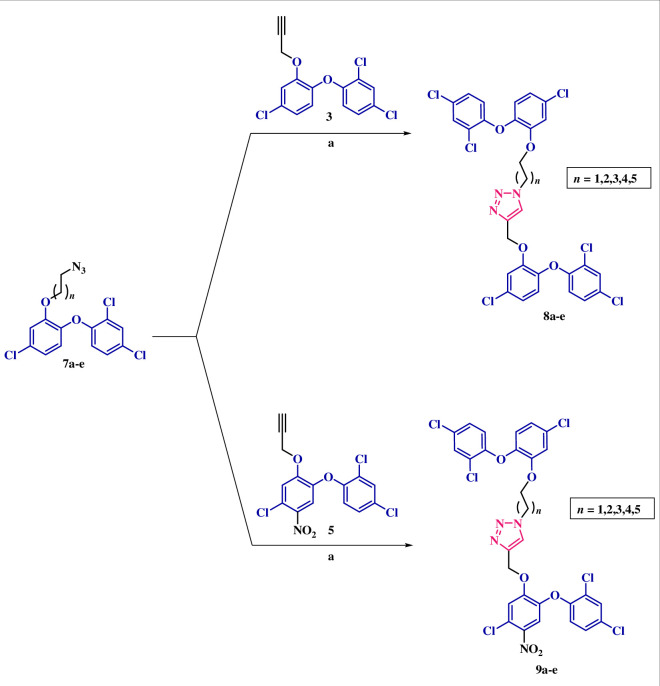
(a) CuI, DIPEA, DMSO, 80°C, 10 min, microwave.

Cu-promoted click reaction [34] of **7a**–**e** with **3** and **5** afforded the corresponding adducts **8a**–**e** and **9a**–**e**, respectively, as shown in Scheme 3. The structures of the synthesized click compounds were assigned based on the spectral data and analytical evidence. For instance, compound **8b** exhibited a molecular ion peak at *m*/*z* C_30_H_21_Cl_6_N_3_O_4_ [M]^+^ 696.9663, [M + 2]^+^ 698.9634, [M + 4]^+^ 700.9604 and [M + 6]^+^ 702.9575 in its high-resolution mass spectrum. The ^1^H NMR spectrum revealed doublets at δ 7.44, 6.95 and 6.90, signifying the presence of aromatic protons derived from TCS. Additionally, two triplets and a multiplet at δ 4.31, 3.92 and 2.28–2.26, respectively, indicated the –CH_2_ alkyl linker groups. Furthermore, the ^13^C NMR spectrum confirmed the proposed structure, displaying the anticipated number of carbons. Notably, characteristic peaks at δ 152.6 and 114.75 were observed for the aromatic carbons of TCS, while peaks at δ 65.09 represented the –CH_2_ connecting the triazole with the oxygen of TCS. The aliphatic carbons of –CH_2_ linker are indicated by the peaks at δ 63.59, 46.48 and 29.54.

The anti-mycobacterial activities of these synthesized triazole-containing TCS dimers (**8a**–**e** and **9a**–**e**) were assessed against *Mabs* CIP104536T and *Mtb* strain mc^2^6230, with TCS, RFB and INH used as reference compounds. The corresponding MIC values are shown in table 2. From these data it can be inferred that all the synthesized triazole tethered dimers in this series are still inactive.

**Table 2.** MIC values determined either in 7H9^OADC/Tylox^ broth against *M. tuberculosis* (*Mtb*) mc^2^6230 at 37°C or in cation-adjusted Mueller–Hinton broth (CaMHB) against *M. abscessus* (*Mabs*) CIP104536^T^ (S variant) at 30°C.

compounds	MIC (µg ml^−1^)	
	*Mabs* S (CaMHB)	*Mtb* (7H9^OADC/Tylox^)
**8a**	>100	>100
**8b**	>100	>100
**8c**	>100	>100
**8d**	>100	>100
**8e**	>100	>100
**9a**	>100	>100
**9b**	>100	>100
**9c**	>100	>100
**9d**	>100	>100
**9e**	>100	>100
TCS	6.25	2.5
INH	>10	0.03
RFB	50	0.19

**Figure 4.**
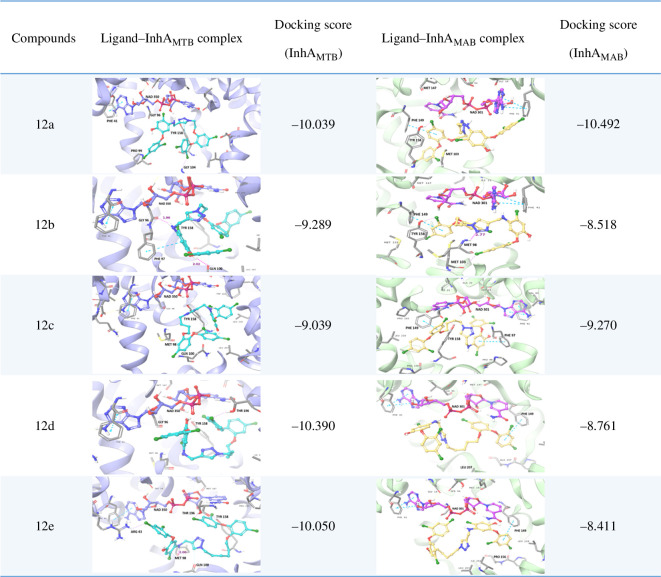
Predicted binding modes of TCS dimers at the active site of InhA_MTB_ (PBD ID: 1P45) and InhA_MAB_ (PBD ID: 7U0M). Residues are shown with grey carbons, NAD^+^ is shown with purple and pink carbons, respectively, dimers are shown with turquoise and yellow carbons, respectively.

In response to the suboptimal outcomes observed in this series, we redesigned triazole-tethered dimers. This redesign involved modifying the position of the propargyl group while maintaining the free status of the –OH group in TCS. Notably, the –OH-substituted ring of TCS exhibits stacking interactions with the nicotinamide ring of NAD^+^ and establishes hydrogen bonds with both the ribose-2-hydroxyl group of NAD^+^ and TYR-158 within the catalytic active site [17]. These compounds, therefore, were strategically designed to occupy the binding sites observed in the PDB: 1P45 and PDB: 7U0M crystal structures. Docking results listed in figure 4 suggested that these compounds exhibited an impressive ability to selectively occupy the active binding sites within both strains. In this series of dimers, the docking scores for InhA_MTB_ ranged from −9.039 to −10.050, significantly surpassing TCS’s docking score of −7.853. The key interactions involved halogen bond interactions between the chlorines of TCS and amino acids GLY-104, GLN-100, THR-196, TYR-158 and ARG-43. Additionally, π*–*π stacking interactions were observed between the triazole and TYR158, as well as between the TCS ring and PHE-197. Two hydrogen bonds were also noted, one formed by the hydroxy group of TCS with GLN-100 and the other by the –NH group with GLY-96. Furthermore, the docking scores ranged from −8.411 to −10.492, once again exceeding TCS’s docking score (−8.309) in the case of InhA_MAB_. Halogen bond interactions were observed with amino acids TYR-158, MET-103 and PRO-156, while π*–*π stacking interactions were found between the TCS rings and PHE-149 and PHE-97. These docking results strongly support the synthesis of these dimers.

**Scheme 4.**
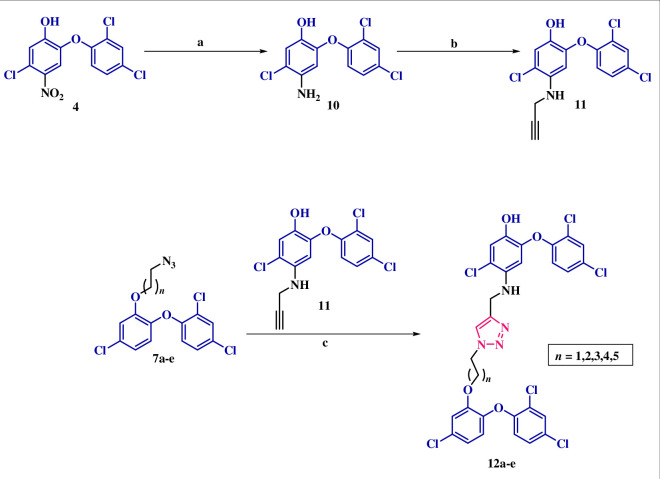
(a) SnCl_2_.2H_2_O, THF, 70°C. (b) Propargyl bromide, K_2_CO_3_, 60°C, 4–5 h. (c) CuI, DIPEA, DMSO, 80°C, 10 min, microwave.

The synthetic approach commenced with the reduction of the nitro-substituted TCS (**4**) using SnCl_2_.2H_2_O to yield the corresponding amine (**10**). Subsequently, **10** underwent base-promoted *N*-propargylation leading to the formation of precursor **11**. Cu-promoted click reaction between the *O*-alkylazido TCS derivatives (**7a**–**e**) with **11** resulted in the formation of the corresponding click adducts (**12a**–**e**), as depicted in Scheme 4. The structures of the synthesized click adducts were assigned based on the spectral data and analytical evidence. For instance, compound **12b** exhibited a molecular ion peak at *m*/*z* C_30_H_22_Cl_6_N_4_O_4_ [M]^+^ 711.9772, [M + 2]^+^ 713.9743, [M + 4]^+^ 715.9713 and [M + 6]^+^ 717.9684 in its high-resolution mass spectrum. Its ^1^H NMR spectrum displayed a singlet at *δ* 5.32, indicating the presence of a (–CH_2_) of propargyl group while another singlet at *δ* 7.50 represented the proton of triazole ring. Additionally, doublets at *δ* 6.71 and 6.89 indicated aromatic protons of TCS. The presence of two triplets and a doublet of triplets at *δ* 4.35, 3.94 and 2.31 indicates the –CH_2_ of alkyl linkers. The ^13^C NMR spectrum further corroborated the assigned structure having all the necessary number of carbons, including characteristic peaks at *δ* 122.15 and 130.73, corresponding to triazole carbons.

In order to study structure–activity relationships of the synthesized triazole tethered *N*-propargylated TCS dimers (**13a**–**e**), they were then evaluated for their anti-mycobacterial activities against the smooth variant of *M. abscessus* (*Mabs*) CIP104536^T^ and *M. tuberculosis* (*Mtb*) strain mc^2^6230 using TCS, RFB and INH as standards, and the MIC values are listed in table 3. As evident, the MIC values are also >100 µg ml^−1^.

**Table 3.** MIC values determined either in 7H9^OADC/Tylox^ broth against *M. tuberculosis* (*Mtb*) mc^2^6230 at 37°C or in cation-adjusted Mueller–Hinton broth (CaMHB) against *M. abscessus* (*Mabs*) CIP104536^T^ (S variant) at 30°C.

compounds	MIC (µg ml^−1^)	
	*Mabs* S (CaMHB)	*Mtb* (7H9^OADC/Tylox^)
**12a**	>100	>100
**12b**	>100	>100
**12c**	>100	>100
**12d**	>100	>100
**12e**	>100	>100
TCS	6.25	2.5
INH	>10	0.03
RFB	50	0.19

The synthesized compounds were then evaluated for their physicochemical and drug-like characteristics, encompassing ADMET (absorption, distribution, metabolism, excretion and toxicity) properties essential for assessing their efficacy and safety. The ADMET descriptor module for small molecules, available at http://www.swissadme.ch/ and http://biosig.unimelb.edu.au/pkcsm/prediction, was employed for ADMET predictions, and the obtained values are presented in electronic supplementary material, table S1. These compounds exhibited favourable characteristics, including good intestinal absorption and safety regarding hepatotoxicity and skin sensitization. Notably, these compounds demonstrated inhibition of several CYP450 isoforms, crucial in drug metabolism and clearance, raising concerns about potential drug–drug interactions and associated adverse effects. Furthermore, all the compounds exhibited increased bioavailability by acting as inhibitors of P-glycoprotein I and II. Additionally, they demonstrated favourable diffusion parameters (Log BB values ranging between −0.078 and −2.287), indicating the potential to cross the blood–brain barrier and minimizing the likelihood of psychotropic side effects.

However, it is noteworthy that the calculated log *P* values, indicative of their permeation through biological membranes, ranged from 8.57 to 12.00, suggesting elevated lipophilicity. This heightened lipophilicity may account for the observed inactivity of these compounds.

## 3. Conclusion

A series of triazole-based dimers were designed and synthesized for assessing their *in vitro* anti-mycobacterial activities against *Mtb* and the non-tuberculous *Mabs* strains. Variation in the nature (alkyl/1H-1,2,3-triazole) as well as position of linker was carried out based on the observed docking score in the binding sites identified in the crystal structures of InhA from *M. tuberculosis* and InhA from *M. abscessus*. The *in vitro* evaluation results, however, revealed that the synthesized compound failed to inhibit the growth of mycobacteria even at the highest tested concentration tested. The higher values of lipophilicity of the synthesized dimers as calculated through ADMET studies could be responsible for their poor activity profiles.

## 4. Experimental

### 4.1. General information

The reactions were performed by employing standard protocols and techniques. Melting points were recorded using open capillaries and Stuart digital melting-point apparatus (SMP10) and were uncorrected. JEOL (400 MHz) and Bruker (500 MHz) spectrometers were used to record ^1^H NMR spectra, and JEOL (100 MHz) and Bruker (125 MHz) spectrometers were used for ^13^C NMR spectra with CDCl_3_ as a solvent. The chemical shifts (*δ*) were expressed in parts per million (ppm) and coupling constants (*J* values) were specified in hertz (Hz). Splitting patterns are designated as s: singlet, d: doublet, t: triplet, m: multiplet, dd: double of doublet, ddd: doublet of doublet of doublet. Using electrospray ionization as the source, mass spectral data were assembled using Bruker high-resolution mass spectrometer (micrOTOF QII) equipment. Microwave reactions were carried out in a microwave synthesis reactor (Anton Paar Monowave 200).

### 4.2. General procedures for the synthesis of compounds

#### 4.2.1. General procedure for the synthesis of compounds **2a**–**e**

The synthesis of the dimers **2a**–**e** involves base-promoted reaction of TCS (**1**) for 10 min to generate the phenolate anion in a microwave reactor followed by the addition of dibromoalkane. The reaction was carried out for 20 min. After usual workup, with ethyl acetate and brine, the purification of each compound was done by column chromatography using ethyl acetate–hexane (0.5 : 9.5) mixture. The resulting desired compound obtained in good yield.

#### 4.2.2. General procedure for the synthesis of 5-chloro-2-(2,4-dichlorophenoxy)-4-nitrophenol (**4**)

A solution of triclosan (7.24 g, 25 mmol) in dichloromethane (20 ml) was stirred with the dropwise addition of 90% nitric acid (1.20 ml). The reaction mixture was stirred at room temperature until the starting material was completely consumed (30 min). After the completion of reaction confirmed by TLC, the reaction mixture was diluted with water (100 ml), extracted with dichloromethane (100 ml × 3) followed by washing with brine (100 ml), saturated solution of sodium bicarbonate (100 ml) and dried with anhydrous sodium sulfate. The drying agent was removed by filtration and the filtrate was evaporated under reduced pressure. The crude product was purified by column chromatography (silica gel, ethyl acetate–hexane 1 : 5). The title compound was obtained as a light-yellow solid.

#### 4.2.3. General procedure for the synthesis of unsubstituted/substituted propargylated triclosan (**3, 5** and **11**)

To well-stirred solutions of **1**, **4** and **10** (1 mmol) separately in acetone, K_2_CO_3_ (1.2 mmol) was added, and the resulting mixture was stirred for 30 min at room temperature to generate anions. On addition of propargyl bromide (1 mmol), the reaction mixture was stirred at 60°C for 12 h. After completion of the reaction as monitored by TLC, the reaction mixture was diluted with water (100 ml), extracted with ethyl acetate (100 ml × 3), washed with water (100 ml), brine (100 ml) and dried with anhydrous sodium sulfate. The drying agent was removed by filtration and the filtrate was evaporated under reduced pressure to yield *O*-propargylated and *N*-propargylated scaffolds in excellent yield.

#### 4.2.4. Procedure for the synthesis of 2-(2-azidoethoxy)-4-chloro-1-(2,4-dichlorophenoxy)benzene (**7a**–**e**)

The procedure for the synthesis of *O*-alkylazido TCS derivatives **7a**–**e** involved an initial base-assisted *O*-alkylation of TCS (**1**) with dibromoalkane in DMF at 100°C for 20 min to yield the corresponding *O*-alkylbromotriclosan **6a**–**e**, followed by subsequent reaction with sodium azide in DMF at 120°C for 10 min. The whole reaction was carried out in a microwave reactor. After the completion of reaction confirmed by TLC, the reaction mixture was diluted with water (100 ml), extracted with dichloromethane (100 ml × 3) followed by washing with brine (100 ml) and dried with anhydrous sodium sulfate. The drying agent was removed by filtration and the filtrate was evaporated under reduced pressure. The crude product was purified by flash chromatography (silica gel, ethyl acetate–hexane 1 : 9). The title compounds were obtained in good yield.

#### 4.2.5. General procedure for the synthesis of 4-amino-5-chloro-2-(2,4-dichlorophenoxy)phenol (**10**)

A mixture of **4** (1.00 g) and tin chloride dihydrate (3.00 g) in dried THF (2.5 ml) was stirred at 70°C for 2 h. After the completion of reaction, as indicated by TLC, the reaction mixture was diluted with ethyl acetate (50 ml), neutralized with a saturated aqueous solution of sodium bicarbonate, extracted with ethyl acetate (50 ml × 2), washed with water (50 ml), brine (50 ml) and dried over sodium sulfate. The drying agent was removed by filtration and filtrate was evaporated under reduced pressure to obtain the desired compound.

#### 4.2.6. General procedure for the synthesis of compounds **8a**–**e, 9a**–**e, 12a**–**e**

The precursors **3/5/11** (1 mmol) and *O*-alkylazidotriclosan derivatives **7a**–**e** (1 mmol) were dissolved in DMSO, CuI (0.143 mmol) was added in catalytic amount and DIPEA (0.143 mmol) was added as base. The reaction mixture was stirred for 10 min at 100°C in a microwave reactor. After the completion of the reaction as monitored by TLC, the crude product was extracted with ethyl acetate and brine and solution of EDTA. The organic layer was dried with sodium sulfate and concentrated under reduced pressure to yield the desired compounds (**8a**–**e**, **9a**–**e**, **12a**–**e**). Compounds were purified by flash chromatography using ethyl acetate–hexane (2 : 8) mixture.

### 4.3. Culture conditions

The pantothenate-auxotrophic *Mtb* mc^2^6230 strain [35] was grown in Middlebrook 7H9 broth supplemented with 10% OADC, 0.025% tyloxapol and 109 μM pantothenic at 37°C without agitation. *Mab* CIP104536^T^ was grown in Mueller–Hinton at 30°C without agitation.

### 4.4. Drug susceptibility testing

MIC was defined as the lowest concentration of compound inhibiting 99% of bacterial growth, at which no change in turbidity was observed and performed in 7H9 broth supplemented with 10% OADC, 0.025% tyloxapol at 37°C (for *Mtb*) without agitation and in CaMHB at 30°C for *Mab*, according to the CLSI guidelines [35]. For *Mtb* mc^2^6230, 109 μM pantothenic acid was added to all experiments. MIC determination was done using the broth dilution method. Briefly, a log-phase (OD_600_ of approx. 1) culture was diluted to OD_600_ = 0.05 (*Mtb*) in 7H9 medium or with an inoculum containing 5 × 10^6^ CFU ml^−1^ (*Mab*) in CaMHB and deposited in 96-well plates. Compounds were then directly added (in general 2 μl per well of a 10 mg ml^−1^ stock solution) to the first-row wells containing 198 µl of bacteria. Serial two-fold dilutions were then completed starting from the first row. Plates were wrapped in parafilm and were then placed in a 37°C incubator and observed after 6 days for *Mtb* or at 30°C and observed after 4 days for *Mab*. Control wells included DMSO as vehicle control, in which bacterial growth was not inhibited (as for untreated wells). INH, TCL and RFB were included as reference drugs.

## Data Availability

Supplementary material is available online [36].
